# Are we forgetting the “universal” in universal masking? Current challenges and future solutions

**DOI:** 10.1017/ice.2020.333

**Published:** 2020-07-16

**Authors:** Sonali D. Advani, Michael E. Yarrington, Becky A. Smith, Deverick J. Anderson, Daniel J. Sexton

**Affiliations:** 1 Department of Medicine, Division of Infectious Diseases, Duke University School of Medicine, Durham, North Carolina; 2 Duke Center for Antimicrobial Stewardship and Infection Prevention, Durham, North Carolina

*To the Editor*—Many US hospitals have recently adopted policies mandating universal masking of all staff, visitors and patients. Universal masking is particularly important in preventing transmission to and from individuals who are asymptomatic or presymptomatic for coronavirus disease 2019 (COVID-19).^1^ The Centers for Disease Prevention and Control (CDC) estimates that ~35% of severe acute respiratory coronavirus virus 2 (SARS-CoV-2) cases are asymptomatic.^2^ In addition, healthcare professionals (HCPs), patients, and visitors with atypical or very mild symptoms may more readily transmit SARS-CoV-2 in healthcare facilities without masking policies.^3^ Risk of exposure to SARS-CoV-2 in nonclinical areas within healthcare facilities may be overlooked.

The incidence of COVID-19 among HCPs decreased significantly after our health system adopted a universal masking policy. Unmasked exposure to another HCP rather than exposure to known infected patients resulted in most of the COVID-19 cases among staff after implementation of this policy.^4^ We recently surveyed 50 community hospitals within the Duke Infection Control Outreach Network and found that 90% of these hospitals had also adopted universal masking policies. However, we also determined that actual compliance with universal masking policies was suboptimal, particularly among staff outside of clinical care settings, including administrative offices, shared work rooms, and break rooms. Poor compliance in these shared spaces led to known exposures in some of these hospitals, leading to employee furloughs, a substantial burden of contact tracing, and unnecessary anxiety for exposed individuals. Here, we discuss our perception and understanding of the etiology of poor compliance with universal masking policies in healthcare settings, and we discuss proposed solutions as well.

## Inaccurate risk perception

Overall, HCP compliance with protective measures such as universal masking often correlates with the level of risk they perceive. Individuals are more likely to comply with recommended prevention measures if they perceive themselves to be at higher risk of harm in a particular situation or setting.^5^ HCPs commonly perceive their risk of contracting COVID-19 from an infected patient to be higher than the risk of exposure to an asymptomatic coworker. Ironically, HCPs spend more time in close proximity to their coworkers than infected patients. A recent study demonstrated that <5% of exposed HCPs tested positive for SARS-CoV-2 despite exposure to an infected patient without adequate personal protective equipment (PPE), although most HCP attribute greater risk to this type of exposure.^6^ The propensity among HCPs to perform inaccurate risk assessments has been seen with other basic infection prevention measures such as hand hygiene.^7^

## Inconsistent messaging from public health authorities

Earlier this year, public health authorities pointed out a lack of evidence related to the use of universal masking by the general public to prevent acquisition of SARS-CoV-2. Later, a member of the World Health Organization (WHO) stated in June 2020 that asymptomatic spread of SARS-CoV-2 is ‘very rare.’ The WHO quickly modified and clarified this statement by stating that asymptomatic spread is incompletely understood even though it actually occurs, contributing to ongoing confusion. Furthermore, a few high-ranking political leaders and millions of citizens routinely ignore the current recommendation to use face coverings in indoor settings and when in close proximity with others. Inconsistent, contradictory and unclear advice from public health authorities has contributed to widespread confusion about the utility of universal masking in preventing the spread of SARS-COV-2 (response efficacy).^5^

The CDC recently updated their exposure guidelines and issued a new “frequently asked question” on May 29, 2019, recommending the use of eye protection when caring for patients in areas of “moderate to substantial community transmission [of SARS-Cov-2],” even if COVID-19 is not suspected.^8,9^ In our opinion, this guidance is confusing and adds an unnecessary emphasis on the use of additional PPE by HCPs when in direct contact with patients and does not place emphasis on the need for universal masking of patients when staff are in close proximity to patients.

## COVID-19 fatigue

COVID-19 fatigue, a term that describes drift in following preventative measures as this pandemic goes on, is an important cause of poor compliance with policies related to universal masking.^10^ This “fatigue” among HCPs may be potentially related to their long work hours, required interactions with other team members throughout the day, the burden of wearing additional eye protection and uncomfortable or poor-quality masks.

## Future strategies related to universal masking

For effective behavioral change, wearing a mask must become a habit for HCPs in all shared spaces inside and outside the workplace, outside of their immediate household and when appropriate physical distancing is not possible.^11^ We need to work closely with HCPs to better understand the root causes for poor masking compliance and to identify and remove barriers to doing the right thing. Simple solutions such as educational campaigns on the rationale for masking, creation of a mask committee comprised of key stakeholders from various worker types to serve as champions, making physical changes to the environment to facilitate distancing, offering better quality masks, as well as suitable and accessable alternate locations that allow for physical separation to occur while HCPs are unmasked during breaks, will likely lead to improved compliance.

Finally, we need clear, simple, and consistent messaging from public health authorities for successful implementation of universal masking policies. Our goal should be to focus on the simple message of universal masking to prevent the transmission of SARS-CoV-2. Healthcare epidemiologists and public health professionals need to learn the art of salesmanship during these times because the message itself, though important, is only as good as the leader that presents it to the public.^12^

## References

[1] Advani SD , Smith BA , Lewis SS , Anderson DJ , Sexton DJ . Universal masking in hospitals in the COVID-19 era: Is it time to consider shielding? Infect Control Hosp Epidemiol 2020 Apr 29 [Epub ahead of print]. doi: 10.1017/ice.2020.179. PMC7218185 32345392

[2] Pandemic planning resources. Centers for Disease Control and Prevention website. https://www.cdc.gov/coronavirus/2019-ncov/hcp/planning-scenarios.html Published 2020. Accessed June 20, 2020.

[3] Klompas M , Morris CA , Sinclair J , Pearson M , Shenoy ES. Universal masking in hospitals in the COVID-19 era. N Engl J Med 2020;382(21):e63. 32237672 10.1056/NEJMp2006372

[4] Seidelman J , Lewis S , Advani S , et al. Universal masking is an effective strategy to flatten the SARS-2-CoV healthcare worker epidemiologic curve. Infect Control Hosp Epidemiol 2020:1–5. 10.1017/ice.2020.313 PMC7520637 32576336

[5] Fakih MG , Sturm LK , Fakih RR. Overcoming COVID-19: addressing the perception of risk and transitioning protective behaviors to habits. Infect Control Hosp Epidemiol 2020 June 9 [Epub ahead of print]. doi: 10.1017/ice.2020.284. PMC7308629 32513323

[6] Baker MA , Rhee C , Fiumara K , et al. COVID-19 infections among HCWs exposed to a patient with a delayed diagnosis of COVID-19. Infect Control Hosp Epidemiol 2020 May 27 [Epub ahead of print]. doi: 10.1017/ice.2020.256. PMC7276498 32456720

[7] Chen LF , Carriker C , Staheli R , et al. Observing and improving hand hygiene compliance: implementation and refinement of an electronic-assisted direct-observer hand hygiene audit program. Infect Control Hosp Epidemiol 2013;34:207–210. 23295569 10.1086/669084 PMC3622086

[8] Potential exposure at work. Centers for Disease Control and Prevention website. https://www.cdc.gov/coronavirus/2019-ncov/hcp/guidance-risk-assesment-hcp.html Published 2020. Accessed June 1, 2020.

[9] Infection control FAQ. Centers for Disease Control and Prevention website. https://www.cdc.gov/coronavirus/2019-ncov/hcp/infection-control-faq.html Published 2020. Accessed June 1, 2020.

[10] Lee LY , Lam EP , Chan CK , et al. Practice and technique of using face mask amongst adults in the community: a cross-sectional descriptive study. BMC Public Health 2020;20:948. 32546228 10.1186/s12889-020-09087-5 PMC7296904

[11] Mergelsberg ELP , Mullan BA , Allom V , Scott A. An intervention designed to investigate habit formation in a novel health behaviour. Psychol Health 2020 Jun [Epub ahead of print]. doi: 10.1080/08870446.2020.1779272. 32546012

[12] Sexton DJ. Hospital epidemiologists and the art of salesmanship. Infect Control Hosp Epidemiol 2018;39:1269–1270. 30165913 10.1017/ice.2018.189

